# Nested ecosystems theory for conceptualizing brain tumors

**DOI:** 10.1242/dmm.052763

**Published:** 2026-02-27

**Authors:** Lori A. Forster, David H. Gutmann

**Affiliations:** Department of Neurology, Washington University School of Medicine, St Louis, MO 63110, USA

## Abstract

The application of advanced multi-omic methodologies to studying brain tumors has culminated in the appreciation that these cancers function as ecosystems that depend on the interactions of a diverse collection of cell types and signals. This connectivity operates not only at the level of the cancer cell, in which variants create new growth dependencies, but also between tumor cells and the immediate tumor microenvironment, between tumor cells and cell populations residing elsewhere in the brain tissue or body, and in response to extracorporeal factors. The cellular and molecular relationships within these four interrelated strata (intracellular, extracellular, intracorporeal and extracorporeal) act in concert to dictate brain tumor development, progression, and therapeutic response by creating biological heterogeneity and unique growth dependencies. In this Perspective, we apply the concept of nested ecosystems to the most common brain tumor (glioma), providing a contextual framework to define how risk factors modify central nervous system oncobiology and to identify future targeted approaches to disease mitigation.

## Teaser

**Summary:** We propose a model of interactive and nested ecosystems to conceptualize how risk factors modify brain tumor pathogenesis, as well as a framework to discover alternative approaches to disease mitigation.

## Introduction

Cancers are cellular communities comprised of numerous distinct cell types (e.g. neurons, glia, immune cells, blood vessels) that profoundly influence the behavior of the cancerous cells. Owing to the innate cellular heterogeneity of these tumors, it is now important to consider new conceptual frameworks that more accurately characterize oncologic disease states relevant to improved therapeutic management strategies. Borrowing from other fields of study, such as ecosystems theory (Bronfenbrenner and Evans, 2000), cancers can be envisioned as ecosystems (Gutmann, 2020), in which approaches, such as network theory, can be employed to test hypotheses and identify promising treatment targets (Douw et al., 2026). In this Perspective article, we propose a holistic model involving ‘nested ecosystems’ to define how risk factors modify brain tumor (glioma) pathogenesis, as well as to discover alternative approaches to disease mitigation. We chose glioma as a case study because they represent one of the most common primary brain tumors in children and adults (Partap and Monje, 2020; Ostrom et al., 2023). Moreover, some of these cancers are difficult to manage using currently available therapies, as treatments can be associated with secondary brain dysfunction (e.g. seizures, cognitive problems). It should be appreciated that although this Perspective focuses on gliomas, it could also be applied as a roadmap for conceptualizing other disorders affecting the brain (e.g. Alzheimer's disease, multiple sclerosis).

… we propose a holistic model involving ‘nested ecosystems’ to define how risk factors modify brain tumor (glioma) pathogenesis, as well as to discover alternative approaches to disease mitigation

## Nested ecosystems

**Fig. 1.**
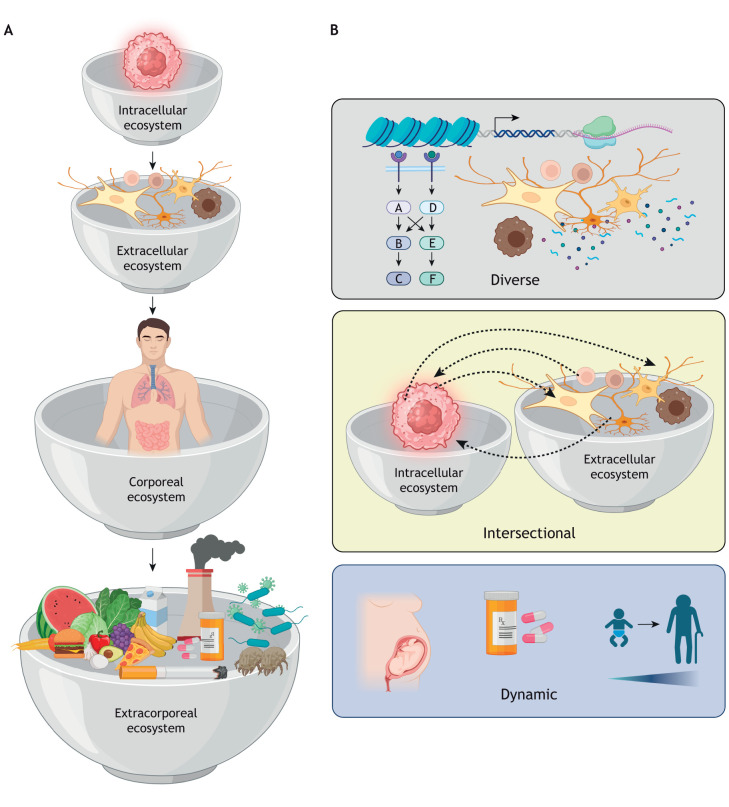
**Reimagining brain tumors as nested ecosystems.** (A) We envision four ecosystems operating at the cellular (intracellular), tissue (extracellular), body (corporeal) and environmental (extracorporeal) levels. The organization of these ecosystems is hierarchical as depicted by the bowls nesting within each other. (B) There are three core characteristics of nested ecosystems: diverse, exhibiting spatial and temporal variability at molecular and cellular levels; intersectional, in which individual signaling programs, transcriptional programs, cells and tissues communicate both within their specific ecosystem and between ecosystems; and dynamic, with the capacity to respond and adapt to changes imposed by extrinsic factors. Created in BioRender by Gutmann, D. H. (2026). https://BioRender.com/5lhnsyt. This figure was sublicensed under CC-BY 4.0 terms.

The idea of interconnected ecological levels, similar to a set of nested Russian dolls, has been used to describe ecological relationships that reflect different levels of organization (Kondoh et al., 2010). Extending this to neuro-oncology, we envision that nervous system tumor biology is dictated by a series of intersecting hierarchical and nested ecosystems. We propose four interconnected ecosystems (strata) that operate at the intracellular, extracellular, intracorporeal and extracorporeal levels (Fig. 1A). The intracellular ecosystem is a self-contained entity in which cell function is controlled by events that operate at the DNA, RNA and protein levels. These include changes in chromatin accessibility, cell cycle progression, RNA transcription and protein translation, as well as signaling pathways involving protein interactions, subcellular compartment trafficking and secondary post-translational modifications. Additionally, energy metabolism (e.g. within the mitochondria) and ion channel function (e.g. at the cell membrane) create different intracellular environments for each of these events, which culminate in distinct biological processes, such as apoptosis. The extracellular ecosystem encompasses the local tumor microenvironment composed of numerous distinct cell types, paracrine signals and cell–stromal interactions that modify intracellular processes within the cancer cells, such as motility and proliferation, through the activation or inhibition of receptor-mediated signaling pathways. Intracorporeal ecosystems are characterized by molecular and cellular interactions between organ systems that can be implemented by circulating hormones or trafficking of immune cells, for instance. Finally, extracorporeal ecosystems are defined by environmental influences, such as viral and bacterial infections.

Importantly, these four ecosystems each possess three core properties (Fig. 1B). First, they exhibit biodiversity, including spatial and temporal variability, which operate at the molecular and cellular level. Second, they are intersectional, such that individual signaling pathways, transcriptional programs, cells and tissues communicate both within and between each specific stratum. Third, they are dynamic and have the capacity to evolve in response to changes imposed by extrinsic factors, akin to the process of ecological succession.

## Intracellular ecosystem

**Fig. 2.**
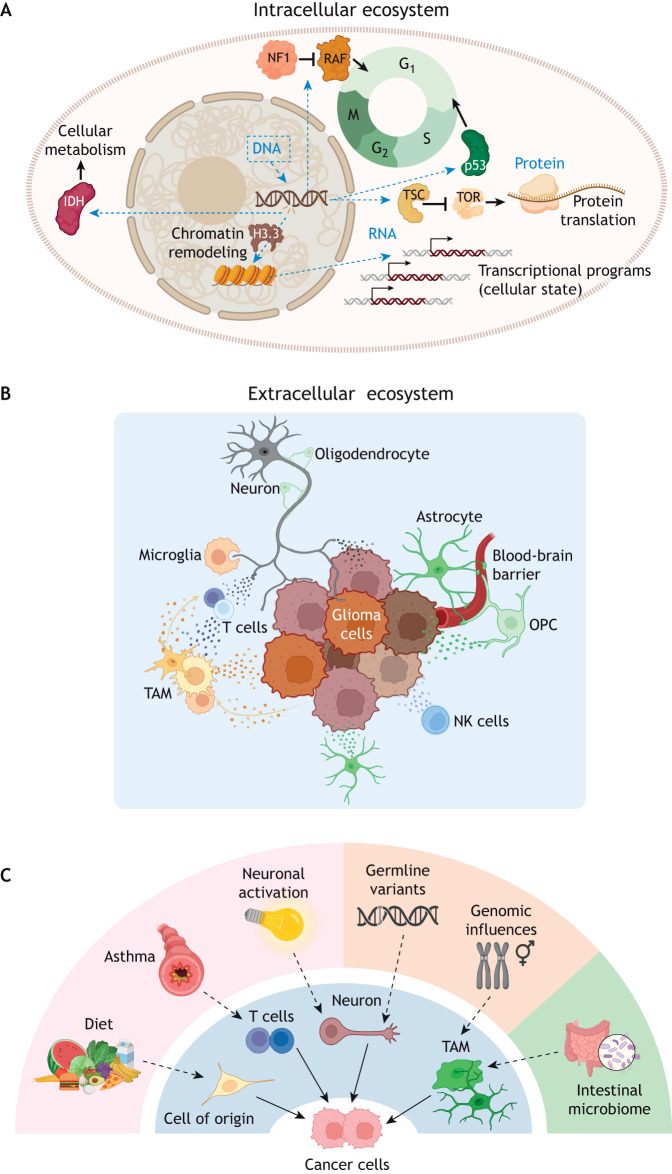
**The four nested ecosystems (strata) function as separate cellular villages with the capacity to interact with each other.** (A) Within the intracellular ecosystem, glioma-associated genomic and genetic alterations can alter chromatin structure, RNA transcription, protein translation and cell metabolism, and influence the function of other processes within the cell (blue dashed line arrows). (B) The extracellular ecosystem is composed of numerous distinct cell types, including tumor cells, immune cells [natural killer (NK) cells], neurons, glia, oligodendroglia lineage cells [oligodendrocytes, oligodendrocyte precursor cells (OPC)] and vasculature (pericytes), which interact in complex circuits to dictate brain tumor biology. In the case of tumor-associated monocytes (TAM), beyond microglia-mediated synaptic pruning, there is reciprocal remodeling of cancer cells, T lymphocytes and tumor-associated monocytes as a result of these interactions. (C) Changes in the intracorporeal (e.g. intestinal microbiome, in green) and extracorporeal (e.g. allergens that cause asthma, in pink) and intracellular (e.g. germline variants, in orange) ecosystems converge on cell types within the extracellular ecosystem (in blue). H3.3, histone-3; IDH, isocitrate dehydrogenase; NF1, neurofibromin 1; p53, tumor protein p53; RAF, rapidly accelerated fibrosarcoma; TOR, target of rapamycin; TSC, tuberous sclerosis complex. Created in BioRender by Gutmann, D. H. (2026). https://BioRender.com/pbyx0wc. This figure was sublicensed under CC-BY 4.0 terms.

The intracellular ecosystem describes the culmination of all processes occurring at the DNA, RNA and protein levels that together establish a particular state of cellular function (Fig. 2A). It is influenced by events operating outside the plasma membrane and could evolve depending on the acquisition of new genomic/genetic alterations, regulation by intracellular feedback circuits or activation/suppression of gene regulatory programs. Gliomas arise as the result of genomic and genetic alterations occurring in single cells, most often disrupting genes or genetic programs important for the normal control of cell growth and differentiation. These include ‘loss-of-function’ variants in genes that negatively regulate cell growth and differentiation (tumor-suppressor genes), ‘gain-of-function’ variants or genomic rearrangements in genes that positively control these processes (oncogenes) and alterations in genes that maintain chromatin stability. In this regard, children with brain cancer predisposition syndromes, such as neurofibromatosis type 1 or Li–Fraumeni syndrome, are born with germline variants in one copy of their respective tumor suppressor gene – either neurofibromin 1 (*NF1*) or tumor protein p53 (*TP53*) – but develop gliomas upon somatic disruption of the remaining normal allele (Gutmann et al., 2000; Sloan et al., 2020). Each of these syndromes represent loss-of-function events involving genes that encode proteins critical for normal brain formation and homeostasis, including proteins in the RAS/ERK mitogenic pathway important for glial cell specification (Li et al., 2012); the mTOR pathway that controls cellular metabolism, RNA transcription and protein translation (Goldberg et al., 2025); and signaling cascades that limit cell cycle progression [e.g. cyclin dependent kinase inhibitor 2A (*CDKN2A*)] (Rahme et al., 2023).

Children who develop gliomas in the absence of an inherited cancer syndrome acquire genetic alterations that lead to gain-of-function variants, aberrantly activating some of these same pathways, such as *QKI:NTRK2* and *KIAA1549:BRAF* genomic rearrangements (Jones et al., 2008) and fibroblast growth factor receptor 1 (*FGFR1*)-activating variants (Zhang et al., 2013). All of these genetic alterations converge on RAS/MAPK mitogenic signaling (Milde et al., 2021), prompting clinical trials using RAS/MEK inhibitors (NCT05804227, NCT04923126, NCT03363217, NCT02285439). In this manner, cancer cells ‘hijack’ normal developmental signaling programs to favor their own proliferation and survival. It should be appreciated that these effects on the intracellular ecosystem are bidirectional, such that cancer cells are both influenced by stromal cells as well as influence their local non-neoplastic extracellular ecosystem.

Beyond genetic or genomic alterations that alter cell growth, other events operate to change the cellular state through the deregulation of key transcription factors important for maintaining terminal differentiation or normal nervous system function (Couturier et al., 2020; Vladoiu et al., 2019). For example, aberrant expression of the oligodendrocyte transcription factor 2 (OLIG2) and SRY-box transcription factor 2 (SOX2) transcription factors (Suva et al., 2014) causes cellular dedifferentiation, creating a ‘stalled’ progenitor-like state, which allows cancer cells to adapt to their changing local microenvironment (Nomura et al., 2025; Dirkse et al., 2019; Neftel et al., 2019). Similarly, cells within the tumor edge exhibit neural precursor cell- and oligodendrocyte precursor cell (OPC)-like properties, displaying transcriptional profiles that mimic those involved in normal neuronal migration (Venkataramani et al., 2022) and neuronal signaling (Varn et al., 2022). Additionally, some tumor cells acquire alterations in the isocitrate dehydrogenase (*IDH1/2*) or histone-3 (H3.3) genes, which disrupt normal metabolic processes or lead to changes in chromatin structure that alter gene expression, cell division and differentiation, respectively (Sturm et al., 2012; Liu et al., 2022). Each of these intracellular changes alters how the cancer cell responds to and adapts to its local microenvironment.

## Extracellular ecosystem

The extracellular ecosystem is composed of the local microenvironment in which a given cancer cell resides, consisting of multiple non-neoplastic cell types, secreted factors and extracellular matrix components that converge on intracellular signal transduction pathways within the intracellular ecosystem. In gliomas, these include tumor-associated monocytes (TAMs), T lymphocytes, neurons, glial lineage cells (e.g. astrocytes, oligodendrocytes) and blood vessels (Fig. 2B).

TAMs are one of the largest non-neoplastic cell populations in brain cancers, representing ∼30-50% of cells in the tumor (Batchu et al., 2023). TAMs include brain-resident microglia and infiltrating peripheral macrophages, with heterogeneous effects on tumor biology owing to the diverse phenotypical identities of these myeloid cells (Yabo et al., 2024). In this regard, TAMs can be cytotoxic to tumor cells (Maximov et al., 2019), or they can increase tumor growth by maintaining glioma stem cell proliferation and self-renewal in glioblastoma (Gu et al., 2025) and by promoting glioma spread within the brain (Ye et al., 2012; Hambardzumyan et al., 2016).

Continuing the tumor-promoting activities, tumor-associated macrophages can contribute to immune suppression, allowing brain cancers to escape T cell-mediated elimination (Gu et al., 2025; De Leo et al., 2024). Additionally, tumor-associated macrophages can support mesenchymal transition, whereby specialized lipid-laden macrophages fuel glioma cell energy demands through the transfer of lipids (Governa et al., 2024; Kloosterman et al., 2024). Like macrophages, tumor-associated microglia have diverse phenotypes in brain tumors (Yabo et al., 2024), contributing to glioma cell invasion and increased tumor growth and proliferation. For example, granulocyte–macrophage colony-stimulating factor (CSF2) secreted by glioma cells can stimulate microglia to increase glioblastoma invasion (Yu-Ju Wu et al., 2020), whereas microglia in experimental models of pediatric low-grade glioma maintain tumor growth through the production of soluble factors (Guo et al., 2020).

In addition to TAMs, T lymphocytes, including CD4^+^ and CD8^+^ T cells, comprise ∼1-5% of glioma cellular content (Chen et al., 2021). Although T cells are not typically resident cells in the brain, they can influence neuron function and contribute to cognition and behavior in mice through the amplification of paracrine factors (Alves de Lima et al., 2020). Of particular interest to oncologists is the CD8^+^ exhausted T cell, which can be reactivated to kill tumor cells (Long et al., 2025). However, the function of this exhausted immune cell population can differ depending on the tumor type or malignancy grade, such that exhausted CD8^+^ T cells in a murine model of low-grade glioma function to increase cell growth (Barakat et al., 2024). The use of immune checkpoint inhibitors that reactivate these exhausted CD8^+^ T cells and facilitate the release of cytotoxic molecules that result in cancer cell death has entered clinical trials for many cancers, including gliomas (NCT03576612, NCT03925246). The appreciation that T cells are key regulators of glioma biology has prompted the investigation of numerous T cell-based therapies, including vaccines to increase T-cell responsiveness and chimeric antigen receptor (CAR) T cells (Monje et al., 2025; Majzner et al., 2022).

Neurons can also regulate brain tumor growth through activity-dependent secretion of paracrine factors, direct synapses with tumor cells and non-synaptic electrical communication. Activity-dependent neuron secretion of paracrine factors can increase glioma cell growth through interactions with other cell types (Pan et al., 2021; Venkatesh et al., 2015, 2017; Anastasaki et al., 2022, 2024). Alternatively, neurons can direct tumor growth through the formation of bona fide synapses with glioma cells. AMPA-receptor dependent synapses (Venkataramani et al., 2019; Venkatesh et al., 2019), GABAergic synapses (Barron et al., 2025) and cholinergic synapses (Drexler et al., 2025; Sun et al., 2025; Tetzlaff et al., 2025) have all been identified in brain tumors. The functional relevance of these synapses is evidenced by their effects on glioma cell proliferation (Venkataramani et al., 2019; Venkatesh et al., 2019; Barron et al., 2025; Drexler et al., 2025; Sun et al., 2025) and invasion (Venkataramani et al., 2022). Additionally, neurons can establish non-synaptic electrical communication with tumor cells through gap junctions in tumor microtubes (Venkatesh et al., 2019). For instance, abrogating electrical or chemical signaling from neurons inhibits glioma growth (Venkatesh et al., 2019), where neuronal activity induces propagating Ca^2+^ transients through tumor microtubes (Venkataramani et al., 2022). Like synaptic activity, this Ca^2+^ activity increases glioma growth (Hausmann et al., 2023). Given this strong preclinical evidence for neuronal signaling regulation of tumor growth, several clinical trials have been initiated (NCT05664464, NCT07284069, NCT04295759).

Glial cells (astrocytes, oligodendrocytes and OPCs) can also influence tumor growth. In malignant gliomas, astrocytes interact with tumor cells to induce T-cell apoptosis (Faust Akl et al., 2025). Astrocytes and tumor cells can also bidirectionally communicate through a network of gap junctions (Venkataramani et al., 2022), leading to reduced inflammasome activation in astrocytes and inhibition of tumor cell necroptosis (Andersen et al., 2025).

Different cell types have been proposed as the cells of origin for glioma experimentally, with neural, glial and oligodendrocyte progenitor cells being capable of forming tumors (Alcantara Llaguno et al., 2015). In gliomas arising from OPCs (Liu et al., 2011; Solga et al., 2017; Rahme et al., 2023), the cell of origin is intriguing, because brain neurons normally communicate with OPCs in the healthy brain to regulate proliferation (Chen et al., 2018a) and direct myelination and oligodendrogenesis through glutamate release (Gibson et al., 2014). In the setting of malignant brain cancer, glutamatergic neuron–glioma synaptic activation increases tumor proliferation (Taylor et al., 2023; Venkatesh et al., 2019; Venkataramani et al., 2022), whereas in low-grade gliomas, glutamate noncanonically activates oncogenic signaling by coupling glutamate receptors to PDGFRα signaling (Anastasaki et al., 2025).

Similarly, other stromal factors can influence cancer cell intracellular signaling. Hypoxia can lead to cellular reorganization within the tumor microenvironment (Greenwald et al., 2024), driving the maintenance of an undifferentiated cellular state (Gustafsson et al., 2005). Additionally, tissue-level mechanical forces can influence tumor cell growth and migration, with increased rigidity favoring enhanced migration (Grundy et al., 2016; Kim et al., 2014). Interestingly, patient survival is inversely correlated with glioma expression of piezo-type mechanosensitive ion channel component 1 (PIEZO1), whereby PIEZO1 activation creates a positive-feedback loop to further increase membrane tension (Chen et al., 2018b).

At the tissue level, these various cell types can organize into spatially distinct subregions (niches), in which the behavior of the tumor cells is dictated by the cellular and molecular constraints unique to that microstructure. The use of spatial transcriptomics and statistical modeling has revealed numerous location-specific cellular interactions relevant to glioma biology (Alcantara Llaguno and Parada, 2021; Kang et al., 2025; Piyadasa et al., 2025). Some of these niches are characterized by hypoxia and the residence of dormant cancer stem cells (Zheng et al., 2021), whereas others are enriched in vascular elements (Pietras et al., 2014) or represent the leading edge of an invasive tumor (Lenzen et al., 2024; Ren et al., 2023; Onubogu et al., 2024; Vishnoi et al., 2026). Although these substructures may be spatially distinct, it is likely that they communicate with each other during tumor evolution and in response to treatment.

## Intracorporeal and extracorporeal ecosystems

Although the brain was once considered an immune-privileged site, sealed off from contact with the body via the blood–brain barrier (BBB), it is now appreciated that there are constant interactions between the central nervous system and other tissues in the body (Smyth and Kipnis, 2025), which we term the intracorporeal ecosystem (Fig. 2C). These modes of communication can involve (1) infiltrating sentinels (e.g. T cells) that enter the healthy brain parenchyma, (2) opportunistic sentinels that invade the brain tissue in the setting of brain pathology (e.g. macrophages), (3) drive-by (passenger) sentinels that do not directly infiltrate the brain parenchyma but rather release soluble factors into the cerebrospinal fluid, and (4) peripheral nervous system feedback to the brain. In contrast, the extracorporeal ecosystem encompasses how the outside world interacts with the body, where the intestinal tract and lungs represent direct portals from the outside world into the body. In this regard, both what we consume and what we breathe can influence brain tumor growth.

Infiltrating sentinel T cells can directly enter the brain through the leptomeningeal or parenchymal blood vessels to interact with resident cell types in the central nervous system (Yoshida et al., 2025). For example, in experimental murine low-grade glioma models, infiltrating T cells that had been exposed to house dust mite or ovalbumin (asthma induction) inhibit TAM support of tumor growth (Chatterjee et al., 2021). The importance of these T cells is illustrated by the protective effect of asthma on brain tumor development in children (Roncarolo and Infante-Rivard, 2012; Porcelli et al., 2016). In this regard, children with asthma exhibit a reduced incidence of brain tumors, and a history of atopy confers a survival benefit in individuals with diffuse low-grade glioma (Jaman et al., 2021; Lehrer et al., 2019). Similarly, allergic airway inflammation delays high-grade glioma progression (Poli et al., 2023).

In the setting of brain disease, the BBB can become compromised, allowing peripheral bone marrow-derived monocytes to enter the brain as opportunistic sentinels. Disruption of the BBB is common in malignant brain tumors, in which infiltrating monocytes are recruited from the blood into the tumor by chemokines [e.g. C-C motif chemokine 2 (CCL2)] (Chang et al., 2016). These monocytes are shaped by tumor-associated variants and brain location, which can positively regulate malignant glioma growth (Ross et al., 2024). As outlined in the section above, TAMs have various functions to enhance glioma progression.

Drive-by sentinel effects have been reported in both health and disease states. For example, meningeal lymphatics communicate with microglia to regulate synaptic function and behavior through paracrine factors [e.g. interleukin (IL)-6] (Kim et al., 2025b), and meningeal T cells produce IL-7 that acts on neurons to regulate anxiety-like behavior (Alves de Lima et al., 2020). Similarly, helper T (TH2) cells communicate with neurons through IL-4 production to reverse experimental multiple sclerosis (allergic encephalomyelitis) progression (Vogelaar et al., 2018) and protect against neuronal injury in mice (Walsh et al., 2015). Additionally, soluble factors influenced by the intestinal microbiome have profound impact on brain function. In low-grade glioma models, intestinal *Bacteroides* stimulates intestinal production of transforming growth factor beta (TGFβ), which enters the circulation to induce TAM recruitment of CD8^+^ T cells to support optic glioma tumor growth (Chatterjee et al., 2025). Furthermore, resident gut *Roseburia faecis* is associated with decreased microglia caspase 3 activation, preventing TAMs from exhibiting anti-glioma properties (Chen et al., 2025).

Leveraging exciting observations in other cancers [e.g. lung cancer (Savchuk et al., 2025)], peripheral neuronal input can also alter the brain immune microenvironment (Amit et al., 2025). This has led to a clinical trial of vagal nerve stimulation as an adjuvant therapy for glioma (Brem, 2024).

The extracorporeal ecosystem can either increase or decrease glioma risk based on the diet consumed. In this respect, a high-salt diet can induce gut dysbiosis and increase glioma growth through increased TGFβ signaling (Kim et al., 2025a), while high-fat, high-sugar diets operate at the level of the cancer cell of origin to increase the incidence and decrease the latency of low-grade gliomas in mice (Chan et al., 2024). Conversely, dietary supplementation with omega-2 fatty acids suppresses glioblastoma initiation by maintaining a quiescent state of mutant neural progenitors (Amodeo et al., 2023), and a ketogenic diet extends survival in mouse glioblastoma models (Mukherjee et al., 2019).

Unfortunately, there is limited evidence implicating other environmental exposures in glioma growth, including cigarette smoking (Holick et al., 2007; Silvera et al., 2006), illicit drug use (Wimberly et al., 2024), *in utero* ambient air toxins (Stalberg et al., 2010; von Ehrenstein et al., 2016), maternal radiation exposure (Koga et al., 2025), traumatic brain injury (Marini et al., 2025) or psychological stress (Wang et al., 2024). In contrast, viral infections may influence glioma prevalence, and varicella zoster virus (VZV) infection decreases low-grade glioma risk (Zhong et al., 2023), whereas low levels of VZV antibodies are associated with poor survival outcomes in adults with high-grade glioma (Guerra et al., 2023).

Finally, time of day can influence cancer biology, to the extent that chronobiology studies attempt to synchronize treatments with circadian rhythms (Zhu et al., 2024; Sancar and Van Gelder, 2021). For example, temozolomide and glucocorticoids have differential effects on glioma cell proliferation when treatment schedules are paired with peak expression of specific clock genes (Gonzalez-Aponte et al., 2025; Slat et al., 2017). Additionally, using dosage schedules based on mathematical modeling of glioma cell kinetic responses increased survival in animal models (Leder et al., 2014).

By adopting a nested ecosystems framework to inform research and therapeutics, it now becomes possible to effectively utilize high-content information to interrogate individual cell effects, as well as the interconnectivity of distinct ecosystem strata

## Leveraging nested ecosystems to inform precision medicine

By adopting a nested ecosystems framework to inform research and therapeutics, it now becomes possible to effectively utilize high-content information to interrogate individual cell effects, as well as the interconnectivity of distinct ecosystem strata. Leveraging multiple ‘-omic’ approaches – such as single-cell assay for transposase-accessible chromatin (ATAC) sequencing, single-cell RNA sequencing, single-cell proteomics and spatial transcriptomics (Rao et al., 2021; Ravi et al., 2022; Ren et al., 2023) – to examine cellular and molecular diversity offers unprecedented opportunities to use advanced machine learning approaches to capture the complex relationships throughout the nested ecosystems that dictate tumor biology (Ding et al., 2025; Pan et al., 2022).

Taken together, the nested ecosystem framework predicts that tumor biology will be dictated by the sum of the cellular and molecular interactions occurring within and between the individual ecosystems. Although the wiring of these relationships may remain the same, the context in which they operate is not static, and changes in children versus adults and in the treatment-naïve brain relative to the brain following chemotherapy can occur. Additionally, risk factors that influence brain tumor development and progression most likely converge on the local circuitry in the tumor cell or its local microenvironment to alter established cellular and molecular interactions necessary for tumorigenesis and growth. In this manner, risk factors may provide new opportunities for disease prognostication [e.g. microbiome composition (Jiang et al., 2024), asthma (Turner et al., 2024; Calboli et al., 2011), obesity (Chan et al., 2024)], where informatic assessments might identify individuals most likely to progress or respond to particular therapies. Based on a more detailed understanding of the mechanisms underlying risk factor modulation of tumor growth, further investigation may also serve to identify critical nodes for therapeutic targeting (Eligator et al., 2025), as well as identify potential adaptive changes within the tumor in response to treatments. For example, diet is part of the extracorporeal ecosystem but can influence specific bacterial species within the intestinal microbiome (intracorporeal ecosystem) to produce soluble factors (e.g. TGFβ) that act on immune system cells, such as TAMs and T cells in the brain (extracellular ecosystem), to increase tumor growth through activation of tumor cell mitogenic signaling pathways (intracellular ecosystem). The intersection of these ‘nested’ ecosystems could lead to the development of predictive tools for brain tumor (in this case optic glioma), such as intestinal bacteria composition, as well as therapies that interrupt immune cell support of optic glioma growth. Lastly, as we envision a future in which treatments are tailored to any given individual malignancy, approaching brain tumors as nested ecosystems pinpoints circuits critical for tumor maintenance, which are each amenable to therapeutic targeting, either singly or in combination. This should lead to the development of more effective treatments and durable outcomes for children and adults with nervous system tumors.
